# Leptin Induces IL-6 Expression through OBRl Receptor Signaling Pathway in Human Synovial Fibroblasts

**DOI:** 10.1371/journal.pone.0075551

**Published:** 2013-09-27

**Authors:** Wei-Hung Yang, Shan-Chi Liu, Chun-Hao Tsai, Yi-Chin Fong, Shoou-Jyi Wang, Yung-Sen Chang, Chih-Hsin Tang

**Affiliations:** 1 Department of Orthopedic Surgery, Taichung Hospital, Department of Health Executive Yuan, Taichung, Taiwan; 2 Department of Nursing, National Taichung University of Science and Technology, Taichung, Taiwan; 3 School of Chinese Medicine, College of Chinese Medicine, China Medical University, Taichung, Taiwan; 4 Graduate Institute of Biotechnology, National Chung Hsing University, Taichung, Taiwan; 5 Institute of Biomedical Sciences, National Chung Hsing University, Taichung, Taiwan; 6 Department of Medicine and Graduate Institute of Clinical Medical Science, China Medical University, Taichung, Taiwan; 7 Department of Orthopedic Surgery, China Medical University Hospital, Taichung, Taiwan; 8 Department of Orthopedic Surgery, Chang-Hua Hospital, Department of Health Executive Yuan, Puhsin, Changhua County, Taiwan; 9 Graduate Institute of Basic Medical Science, China Medical University, Taichung, Taiwan; 10 Department of Pharmacology, School of Medicine, China Medical University, Taichung, Taiwan; 11 Department of Biotechnology, College of Health Science, Asia University, Taichung, Taiwan; Virgen Macarena University Hospital, School of Medicine, Spain

## Abstract

**Background:**

Leptin, an adipocyte-secreted hormone that centrally regulates weight control, may exert proinflammatory effects in the joint, depending on the immune response. Leptin is abundantly expressed in osteoarthritis (OA) cartilage and synovium. However, the relationship between leptin and interleukin-6 (IL-6) in OA synovial fibroblasts (OASFs) remains obscure.

**Methodology/Principal Findings:**

Stimulation of OASFs with leptin induced IL-6 expression in a concentration- and time-dependent manner. OASFs expressed the long (OBRl) and short (OBRs) isoforms of the leptin receptor. However, OBRl, but not OBRs, antisense oligonucleotide (AS-ODN) abolished the leptin-mediated increase of IL-6 expression. Transfection with insulin receptor substrate (IRS)-1 siRNA decreased leptin-induced IL-6 production. In addition, pretreatment of cells with PI3K, Akt, or AP-1 inhibitor also inhibited the potentiating action of leptin. Leptin-induced AP-1 activation was inhibited by OBRl, IRS-1, PI3K, or Akt inhibitors and siRNAs.

**Conclusions/Significance:**

Our results showed that leptin activates the OBRl receptor, which in turn activates IRS-1, PI3K, Akt, and AP-1 pathway, leading to up-regulation of IL-6 expression.

## Introduction

Osteoarthritis (OA) is the most common adult joint disease, and is increasing in frequency and severity [1]. The cause of OA is not yet fully understood. However, obesity, inflammation of the joints, repetitive motion, and genetic predisposition are considered to contribute to excessive loading of the joints, leading to the onset of the disease [2]. In response to the proinflammatory mediators produced by chondrocytes and macrophages, osteoarthritic synovial fibroblasts (OASFs) produce cytokines that promote cartilage degradation, neovascularization, and inflammation [3].

The development and progression of OA are now believed to involve inflammation [4], and elevated levels of cytokines, such as interleukin-6 (IL-6) also seems to be the main proinflammatory cytokine involved in the pathophysiology of OA [3]. IL-6 has numerous biological activities and is considered as the major player that regulates the innate immune response, haemopoiesis, and inflammation [5,6]. A previous report showed the concentration of IL-6 to be increased in sera and synovial fluid from OA patients [7]. Combination treatment with IL-1β and oncostatin was found to up-regulate IL-6, MMP-1, and MMP-13 in human cartilage [8,9]. In addition, mechanical injury could potentiate the effects of IL-6 on proteoglycan degradation [10], while treatment of chondrocytes with IL-6 reduced the expression of type II collagen [11]. A clinical trial in OA patients showed that IL-6 was associated with an risk of cartilage loss [12]. These findings strongly indicate an important role of IL-6 production during OA pathogenesis.

Leptin, a small (16-kDa) nonglycosylated peptide hormone encoded by the obese (ob) gene [13], is produced predominantly in white adipose tissue [14]. Leptin is an anorexic peptide that is primarily known for its role as a hypothalamic modulator of food intake, body weight, and fat stores [15]. The biological activity of leptin is mediated by specific receptors (OBR), which are located in several tissues throughout the body [16]. At least 6 isoforms of OBR are generated by alternative messenger RNA splicing, but in humans, 2 major forms of leptin receptor are expressed. The long form of the receptor (OBRl), which is believed to be the signaling-competent receptor isoform, is essential in mediating most of the biological effects of leptin [17]. The signaling pathways activated by OBRl include the classic cytokine JAK2/STAT3 pathway as well as the insulin receptor substrate (IRS)-1/PI3K/Akt pathway [18].

The potential role of leptin in OA is supported by the relationship between high body mass index and an increased risk of OA [19]. Leptin has been detected also in synovial fluid obtained from patients with OA [20,21]. In experimental models, leptin may display proinflammatory effects in the joint, depending on the immune response [22,23]. Therefore, leptin can be easily considered as having a prototypical proinflammatory and catabolic role in cartilage metabolism and progression of OA. Nevertheless, the current understanding of the role of leptin in synovial fibroblasts and OA progression is still incomplete. Here, we explored the signaling pathway involved in leptin-induced IL-6 production in human OASFs. The results showed that leptin activates the OBRl receptor, which in turn activates IRS-1, PI3K, Akt, and AP-1 pathway, leading to up-regulation of IL-6 expression.

## Materials and Methods

### Materials

Rabbit polyclonal antibody specific for p-IRS-1 was purchased from Cell Signaling and Neuroscience (Danvers, MA). Anti-mouse and anti-rabbit IgG-conjugated horseradish peroxidase and rabbit polyclonal antibodies specific for β-actin, IRS-1, p-p110, p110, p-Akt, Akt, p-c-Jun, and c-Jun were purchased from Santa Cruz Biotechnology (Santa Cruz, CA) The recombinant human leptin and IL-6 ELISA kit were purchased from PeproTech (Rocky Hill, NJ). The AP-1 luciferase plasmid was purchased from Stratagene (La Jolla, CA). Curcumin was purchased from Biomol (Butler Pike, PA). The human IL-6 promoter construct pIL6-luc651(−651/+1), AP-1 site mutation (pIL6-luc651ΔAP-1), NF-κB site mutation (pIL6-luc651ΔNF-κB), and C/EBP-β site mutation (pIL6-luc651ΔC/EBP-β) were provided by Dr. O. Eickelberg (Department of Medicine II, University of Giessen, Giessen, Germany). The pSV-β-galactosidase vector and luciferase assay kit were purchased from Promega (Madison, WI). All other chemicals were purchased from Sigma-Aldrich (St. Louis, MO).

### Cell culture

The study was approved by the Institutional Review Board of China Medical University Hospital, and informed written consent was obtained from patients. Human synovial fibroblasts were isolated by collagenase treatment of synovial tissue samples obtained from 8 patients with OA during knee replacement surgeries (Patient information as shown in Table S1). OASFs were isolated, cultured, and characterized as previously described [24,25]. Cells were amplified during 1 to 3 passages, and the experiments were performed using cells from passages 3 to 6.

### Transfection of siRNAs

ON-TARGETplus siRNAS targeting IRS-1, p110, Akt, c-Jun, and control were purchased from Dharmacon Research (Lafayette, CO). Transient transfection of siRNAs was carried out using DharmaFECT1 transfection reagent. siRNA (100 nM) was formulated with DharmaFECT1 transfection reagent according to the manufacturer’s instructions.

### Quantitative real-time PCR

Total RNA was extracted from synovial fibroblasts by using a TRIzol kit (MDBio Inc., Taipei, Taiwan). The reverse transcription reaction was performed using 2 µg of total RNA, which was reverse-transcribed into cDNA using oligo(dT) primer [26]. Quantitative real-time PCR (qPCR) analysis was carried out using TaqMan One-Step PCR Master Mix (Applied Biosystems, Foster City, CA). cDNA templates (2 µl) were added per 25-µl reaction with sequence-specific primers and TaqMan probes. Sequences for all target gene primers and probes were purchased commercially (β-actin was used as an internal control) (Applied Biosystems). The qPCR assays were carried out in triplicate on a StepOnePlus sequence detection system. The cycling conditions involved 10-min polymerase activation at 95 °C, followed by 40 cycles at 95 °C for 15 s and 60 °C for 60 s. The threshold was set above the non-template control background and within the linear phase of the target gene amplification to calculate the cycle number at which the transcript was detected (denoted C_T_).

### Western blot analysis

Cellular lysates were prepared as described previously [27]. Proteins were resolved on SDS-PAGE and transferred onto Immobilon polyvinyldifluoride (PVDF) membranes. The blots were blocked with 4% BSA for 1 h at room temperature and then probed with rabbit anti-human antibodies against IRS-1, p110, Akt, or c-Jun (1:1000) for 1 h at room temperature. After 3 washes, the blots were subsequently incubated with donkey anti-rabbit peroxidase-conjugated secondary antibody (1:3000) for 1 h at room temperature. The blots were visualized by enhanced chemiluminescence with Kodak X-OMAT LS film (Eastman Kodak, Rochester, NY).

### Measurement of IL-6 production

Human synovial fibroblasts were cultured in 24-well culture plates. At confluence, cells were treated with leptin and then incubated in a humidified incubator at 37°C for 24 h. For examination of the downstream signaling pathways involved in leptin treatment, cells were pretreated with various inhibitors for 30 min before leptin (3 µM) administration. After incubation, the medium was removed and stored at −80°C until assay. IL-6 in the medium was assayed using the IL-6 enzyme immunoassay kits according to the procedure described by the manufacturer [24,28].

### Transfection and reporter gene assay

Human synovial fibroblasts were cotransfected with 0.8 µg of luciferase plasmid and 0.4 µg of β-galactosidase expression vector. OASFs were grown to 80% confluence in 12-well plates and were transfected on the following day with Lipofectamine 2000 (LF2000; Invitrogen). DNA and LF2000 were premixed for 20 min and then applied to the cells. After 24 h of transfection, the cells were incubated with the indicated agents. After further 24-h incubation, the media were removed and cells were washed once with cold PBS. To prepare lysates, 100 µl of reporter lysis buffer (Promega, Madison, WI) was added to each well, and cells were scraped from the dishes. The supernatant was collected after centrifugation at 13,000 rpm for 2 min. Aliquots of cell lysates (20 µl) containing equal amounts of protein (20–30 µg) were placed into wells of an opaque black 96-well microplate. An equal volume of luciferase substrate was added to all samples, and luminescence was measured using a microplate luminometer. The luciferase activity was normalized to the transfection efficiency monitored by the cotransfected β-galactosidase expression vector.

### Synthesis of OBRl and OBRs decoy oligonucleotide

We used a phosphorothioate double-stranded decoy oligonucleotide (ODN) carrying the OBRl antisense ODN (AS-ODN; AGACCGAGCGGGCGTTAA) and missense ODN (MM-ODN; AGCCCGCGCGAGTGTTCA) (GenBank accession no. U43168) and the OBRs AS-ODN (TTGTCTTGCCGACCACCA) and MM-ODN (TTATCTTACCAACCGCCA) (GenBank accession no. U50748). ODN (5 µM) was mixed with LF2000 (10 µg/ml) for 30 min at room temperature, and the mixture was added to cells in serum-free medium. After 24 h of transient transfection, the cells were used for the following experiments.

### Chromatin immunoprecipitation assay

Chromatin immunoprecipitation analysis was performed as described previously [28]. DNA immunoprecipitated with c-Jun antibody was purified and extracted with phenol-chloroform. The purified DNA pellet was subjected to PCR. PCR products were then resolved by 1.5% agarose gel electrophoresis and visualized with UV light. The primers 5′-GAACTGACCTGACTTACATA-3′ and 5′-TTGAGACTCATGGGAAAATCC-3′ were used to amplify the human IL-6 promoter region containing the AP-1 binding site (−312 to −39).

### Statistical Analysis

Data were expressed as means ± S.E. Statistical analysis was performed with GraphPad Prism 4. Analysis of variance (ANOVA) and unpaired 2-tailed Student’s *t* test were used to determine the significant differences between the means. *p* < 0.05 was considered significant.

## Results

### Leptin induces IL-6 expression in human synovial fibroblasts through OBRl receptor

**Figure 1:**
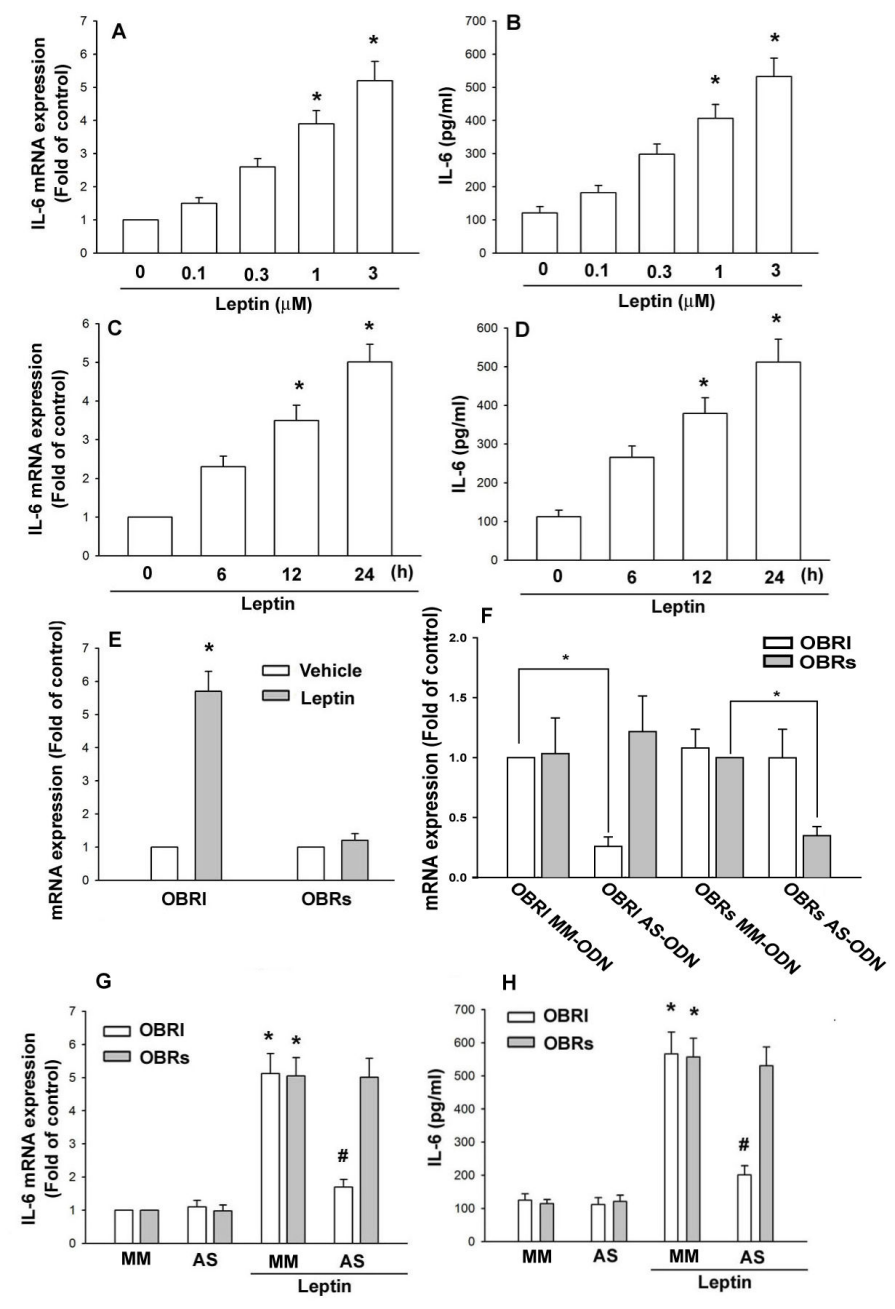
Leptin induces IL-6 expression in human synovial fibroblasts through OBRl receptor. (A and B) OASFs were incubated with various concentrations of leptin for 24 h. Media and total RNA were collected, and the expression of IL-6 was examined by qPCR and ELISA assay (n=4). (C and D) OASFs were incubated with leptin (3 µM) for 6, 12, or 24 h. Media and total RNA were collected, and the expression of IL-6 was examined by qPCR and ELISA assay (n=4). (E) OASFs were incubated with leptin (3 µM) for 24 h. OBRl and OBRs mRNA expression was examined by qPCR (n=4). (F) OASF cells were transfected with OBRl and OBRs AS-ODN or OBRl and OBRs MM-ODN, and mRNA level of OBRl and OBRs was analyzed by qPCR (n=4). (G and H) OASFs were transfected with OBRl and OBRs AS-ODN or OBRl and OBRs MM-ODN for 24 h and then stimulated with leptin (3 µM) for 24 h; IL-6 expression was examined by qPCR and ELISA assay (n=4). Results are expressed as mean ± S.E.M. of four independent experiments. *: *p* < 0.05 as compared with basal level. #: *p* < 0.05 as compared with the leptin-treated group.

Leptin is significantly higher in synovial fluid of patients with OA and rheumatoid arthritis (RA) [29,30]. It has been also reported that leptin plays an important role during OA pathogenesis [31]. Therefore, we used human synovial fibroblasts to investigate the signaling pathways of leptin in the production of IL-6 (an important inflammatory cytokine). Treatment of OASFs with leptin (0.1–3 µM) for 24 h induced IL-6 mRNA and protein expression in a concentration-dependent manner, as shown by qPCR and ELISA assay (Figure 1A and B). In addition, leptin also increased IL-6 mRNA and protein expression dose-dependently (Figure 1C and D). These data indicate that leptin increased IL-6 expression in human OASFs. Leptin increased IL-6 expression to a similar extent as IL-1β (Figure S1). Further work is needed to determine whether leptin has an equal effect in vivo as that shown for IL-1β previously. Previous studies have shown that leptin exerts their cell functions through interaction with specific leptin receptors OBRl and OBRs [32]. We therefore hypothesized that leptin receptors may be involved in leptin-mediated IL-6 expression in OASFs. Treatment of OASFs with leptin for 24 h significantly increased the mRNA levels of OBRl, whereas OBRs receptor mRNA remained unchanged (Figure 1E). We next examined whether leptin receptors are involved in the leptin-mediated increase of IL-6 production. Transfection with OBRl or OBRs antisense oligonucleotide (AS-ODN) specifically inhibited OBRl or OBRs expression, respectively (Figure 1F). In addition, OBRl AS-ODN, but not with OBRl MM-ODN, OBRs AS-ODN, or OBRs MM-ODN, abolished the leptin-induced IL-6 production (Figure 1G and H). Therefore, OBRl receptor plays a key role in leptin induced IL-6 expression in OASFs.

### The IRS-1 and PI3K signaling pathways are involved in leptin-mediated increase of IL-6 expression

**Figure 2:**
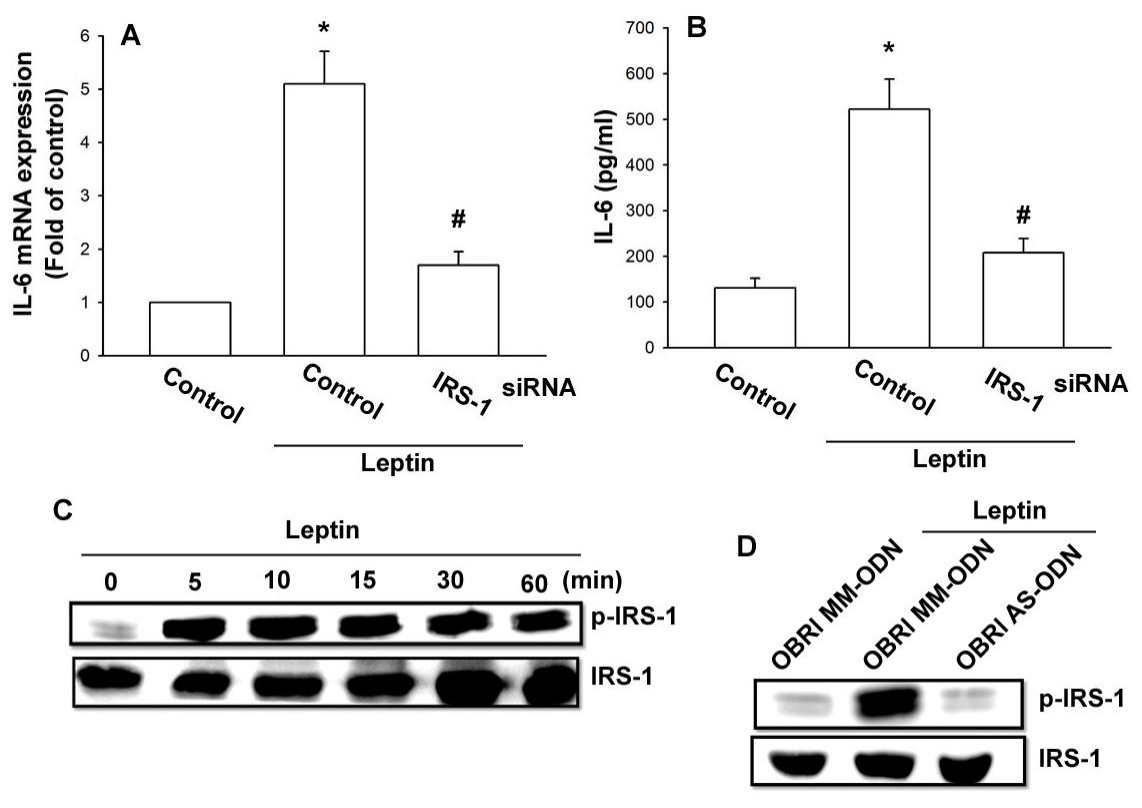
The IRS-1 signaling pathway is involved in leptin-mediated increase of IL-6 expression. (A and B) OASFs were transfected with IRS-1 siRNA for 24 h. Media and total RNA were collected, and the expression of IL-6 was examined by qPCR and ELISA assay (n=4). (C) OASFs were incubated with leptin for the indicated time intervals. Total protein was collected and the phosphorylation of IRS-1 was examined by western blotting assay (n=4). (D) OASFs were transfected with OBRl AS-ODN and MM-ODN for 24 h and then stimulated with leptin (3 µM) for 30 min. Total protein was collected and the phosphorylation of IRS-1 was examined by western blotting (n=4). Results are expressed as mean ± S.E.M. of four independent experiments. *: *p* < 0.05 as compared with basal level. #: *p* < 0.05 as compared with the leptin-treated group.

**Figure 3:**
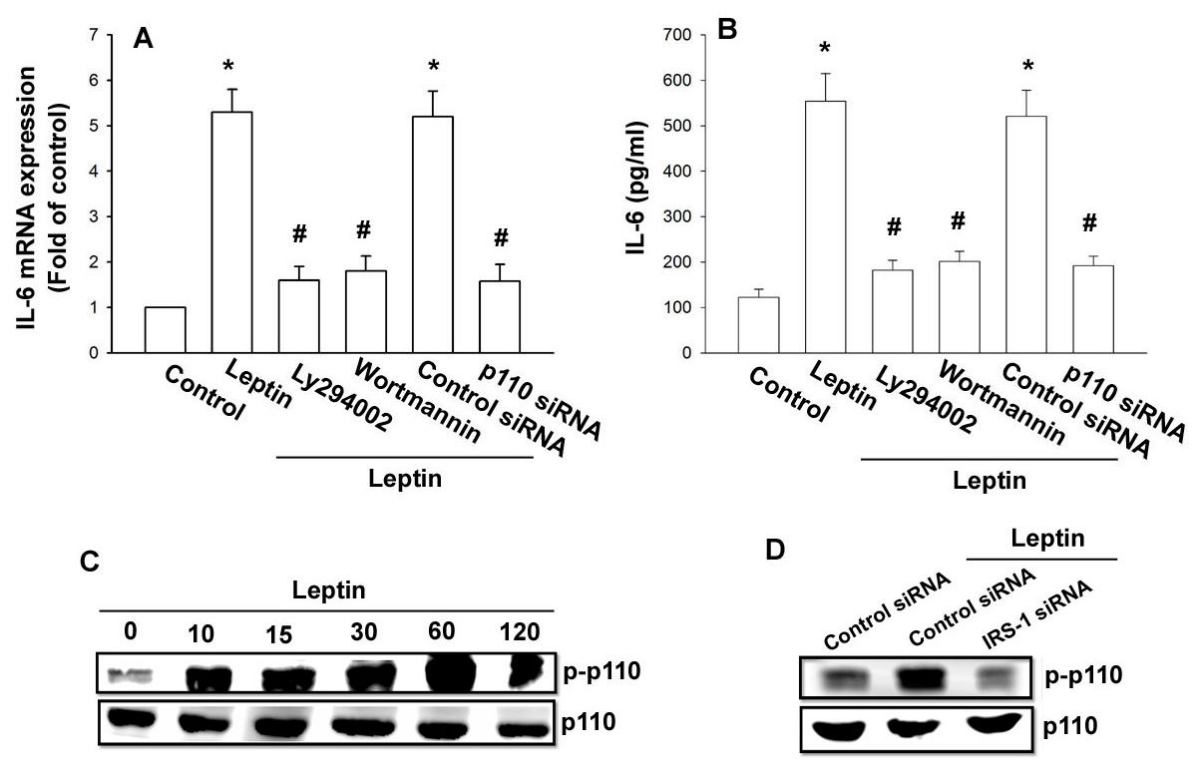
The PI3K signaling pathway is involved in leptin-mediated increase of IL-6 expression. (A and B) OASFs were pretreated for 30 min with Ly294002 and wortmannin or transfected with p110 siRNA for 24 h, followed by stimulation with leptin for 24 h. Media and total RNA were collected, and the expression of IL-6 was examined by qPCR and ELISA assay (n=4). (C) OASFs were incubated with leptin for the indicated time intervals. Total protein was collected and the phosphorylation of p110 was examined by western blotting (n=4). (D) OASFs were transfected with IRS-1 siRNA for 24 h and then incubated with leptin (3 µM) for 60 min; Total protein was collected and the phosphorylation of p110 was examined by western blotting (n=4). Results are expressed as mean ±S.E.M. of four independent experiments. *: *p* < 0.05 as compared with basal level. #: *p* < 0.05 as compared with the leptin-treated group.

Previous studies have shown that leptin induced IRS-1-associated PI3K activity to regulate cell functions [33–35]. To identify whether IRS-1 is involved in leptin-triggered IL-6 production, we used IRS-1 siRNA. Transfection of OASFs with IRS-1 siRNA specifically reduced leptin-induced IL-6 expression (Figure 2A and B). To directly confirm the crucial role of IRS-1 in IL-6 production, we then directly measured the phosphorylation of IRS-1 in response to leptin. Treatment of OASFs with leptin significantly increased IRS-1 phosphorylation (Figure 2C). Transfection of cells with OBRl AS-ODN, but not with OBRl MM-ODN, reduced the leptin-induced IRS-1 phosphorylation (Figure 2D). These results show that leptin seems to act through an OBRl- and IRS-1-dependent signaling pathway to enhance IL-6 production in human synovial fibroblasts. We next examined whether PI3K, a critical downstream target of IRS-1 [35], is involved in leptin-triggered IL-6 production in OASFs. Pretreatment of synovial fibroblasts with specific PI3K inhibitors Ly294002 and wortmannin for 30 min or transfection with p110 siRNA for 24 h markedly decreased the leptin-induced IL-6 production (Figure 3A and B). We then directly measured the phosphorylation of PI3K in response to leptin. Indeed, treatment of fibroblasts with leptin resulted in a time-dependent phosphorylation of p110 (Figure 3C). Furthermore, transfection of cells with IRS-1 siRNA reduced the leptin-induced p110 phosphorylation (Figure 3D). Taken together, these results indicate that leptin increased IL-6 expression in human OASFs via the IRS-1 and PI3K signaling pathway.

### The Akt signaling pathway is involved in the potentiating action of leptin

**Figure 4:**
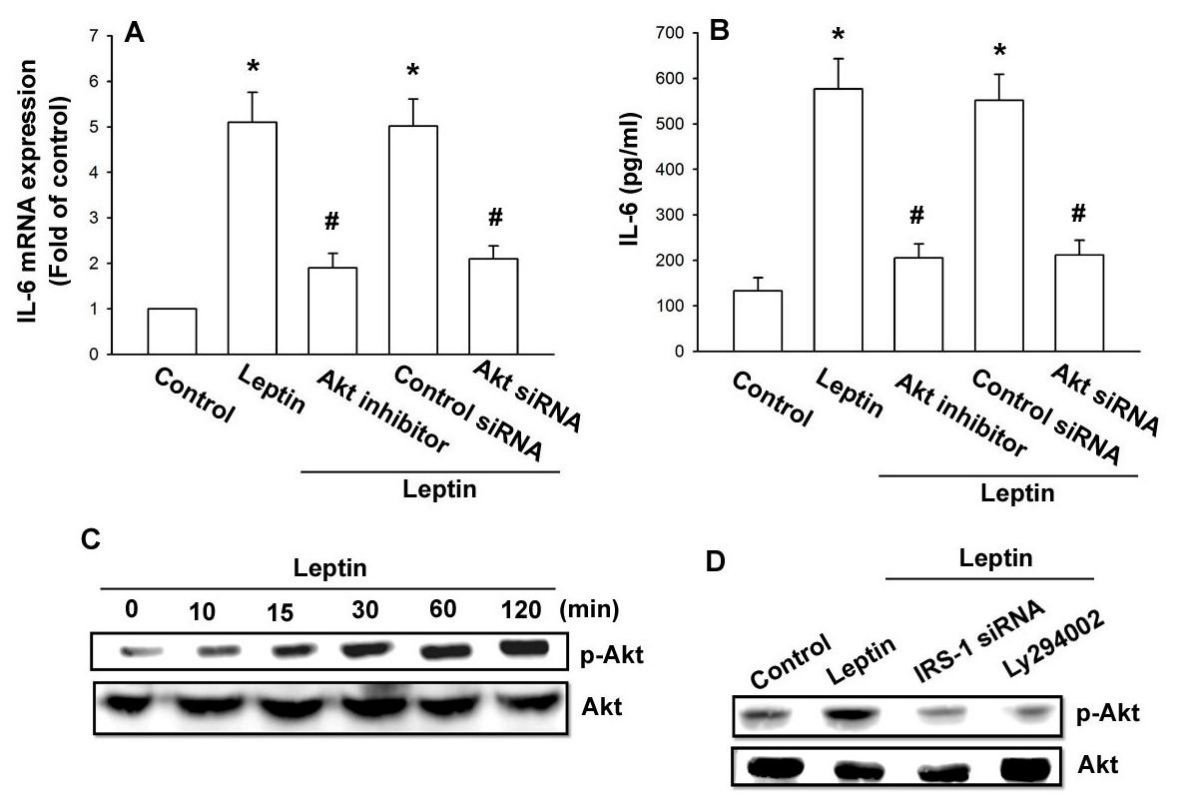
The Akt signaling pathway is involved in the potentiating action of leptin (A and B) OASFs were pretreated for 30 min with Akt inhibitor or transfected with Akt siRNA for 24 h, followed by stimulation with leptin for 24 h. Media and total RNA were collected, and the expression of IL-6 was examined by qPCR and ELISA assay (n=4). (C) OASFs were incubated with leptin for the indicated time intervals. Total protein was collected and the phosphorylation of AKT was examined by western blotting (n=4). (D) OASFs were pretreated for 30 min with Ly294002 or transfected with IRS-1 siRNA for 24 h and then incubated with leptin (3 µM) for 60 min; Total protein was collected and the phosphorylation of AKT was examined by western blotting (n=4). Results are expressed as mean ±S.E.M. of four independent experiments. *: *p* < 0.05 as compared with basal level. #: *p* < 0.05 as compared with the leptin-treated group.

The serine/threonine kinase Akt, a downstream effector of PI3K, is involved in the regulation of cell growth, differentiation, adhesion, and inflammatory reactions [36]. We thus investigated the role of Akt in mediating leptin-induced IL-6 expression by using the Akt inhibitor and Akt siRNA. As shown in Figure 4A and B, leptin-induced IL-6 expression was markedly decreased by pretreatment of cells for 30 min with Akt inhibitor or transfection of cells with Akt siRNA for 24 h. Furthermore, stimulation of OASFs with leptin resulted in time-dependent phosphorylation of Akt (Figure 4C). We next evaluated the relationship between IRS-1/PI3K and Akt in the leptin-mediated signaling pathway, and found that transfection of cells with IRS-1 siRNA or pretreatment of cells with Ly294002 reduced the leptin-induced Akt phosphorylation (Figure 4D). These results indicate that leptin appears to act via the IRS-1, PI3K, and Akt signaling pathway to enhance IL-6 production in human OASFs.

### Involvement of AP-1 in leptin induced IL-6 production

**Figure 5:**
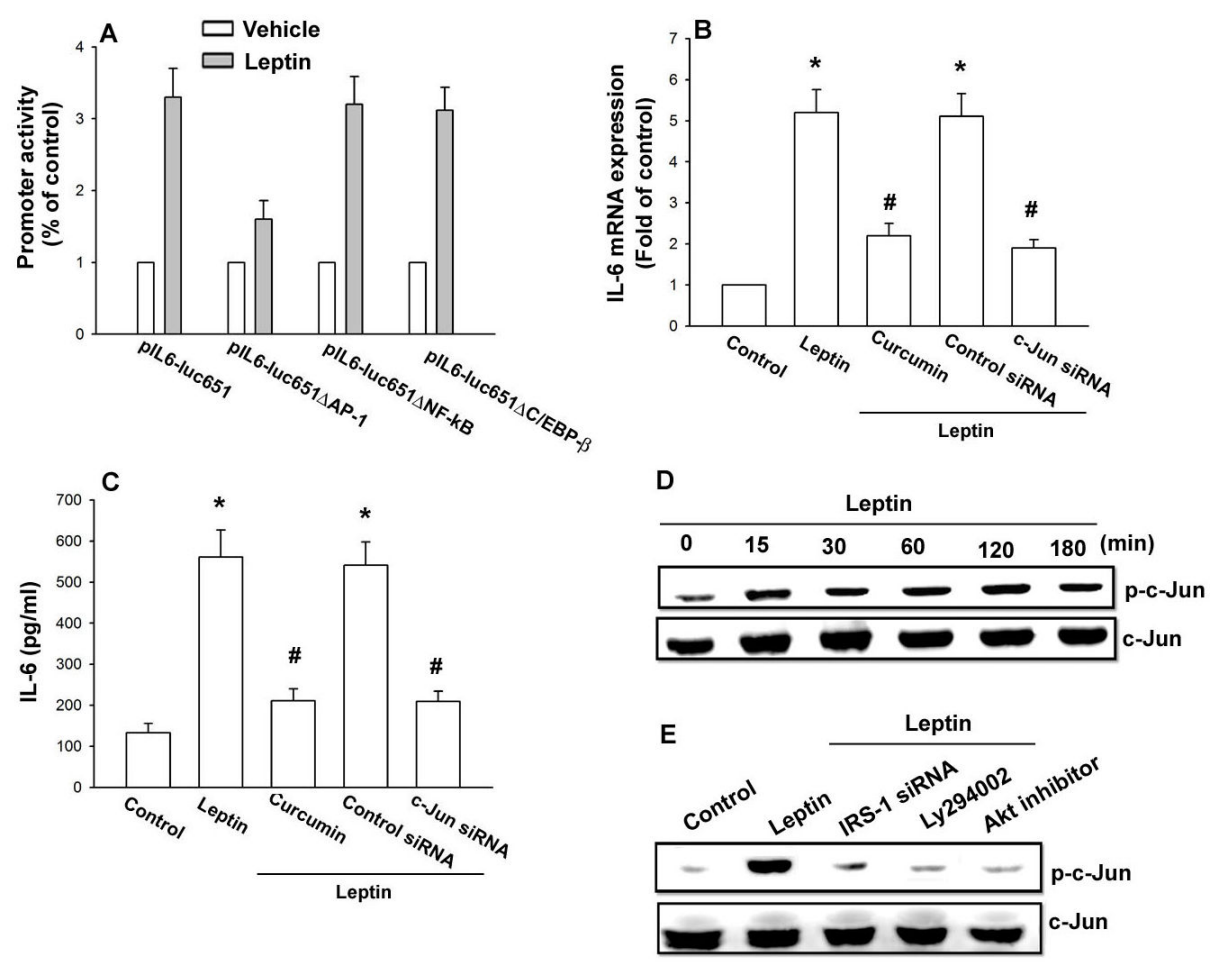
Involvement of AP-1 in leptin induced IL-6 production. (A) OASFs were transfected with indicated IL-6 promoter luciferase plasmids for 24 h. Cells were then incubated with leptin (3 µM) for 24 h. IL-6 luciferase activity was measured, and the results were normalized to β-galactosidase activity and expressed as the mean ± SE for 3 independent experiments performed in triplicate (n=4). (B and C) OASFs were pretreated for 30 min with curcumin or transfected for 24 h with c-Jun siRNA, followed by stimulation with leptin for 24 h. Media and total RNA were collected, and the expression of IL-6 was examined by qPCR and ELISA assay (n=4). (D) OASFs were incubated with leptin for the indicated time intervals. Total protein was collected and the phosphorylation of c-Jun was examined by western blotting (n=4). (E) OASFs were pretreated with Ly294002 and Akt inhibitor for 30 min or transfected with IRS-1 siRNA for 24 h and then incubated with leptin for 120 min. Total protein was collected and the phosphorylation of c-Jun was examined by western blotting (n=4). Results are expressed as mean ±S.E.M. of four independent experiments. *: *p* < 0.05 as compared with basal level. #: *p* < 0.05 as compared with the leptin-treated group.

There are three known *cis*-regulatory elements such as NF-κB, C/EBP-β, and AP-1 in the promoter region of IL-6 gene [37,38]. We examined the role of the transcriptional binding sites in induction of IL-6 expression. Three different IL-6 promoter constructs (pIL6-luc651ΔNF-κB, pIL6-luc651ΔC/EBP-β, and pIL6-luc651ΔAP-1) were generated by site-directed mutagenesis and testing them in a luciferase reporter assay. We found that leptin-stimulated luciferase activity was abolished by AP-1 binding site mutation, but not by NF-κB or C/EBP-β site mutation (Figure 5A). The increase in IL-6 expression mediated by leptin was antagonized by AP-1 inhibitor (curcumin) and c-Jun siRNA (Figure 5B and C). Furthermore, stimulation of OASFs with leptin time-dependently increased c-Jun phosphorylation (Figure 5D). Pretreatment of cells with Ly294002 and Akt inhibitor or transfection of cells with IRS-1 siRNA markedly inhibited the leptin-induced c-Jun phosphorylation (Figure 5E).

**Figure 6:**
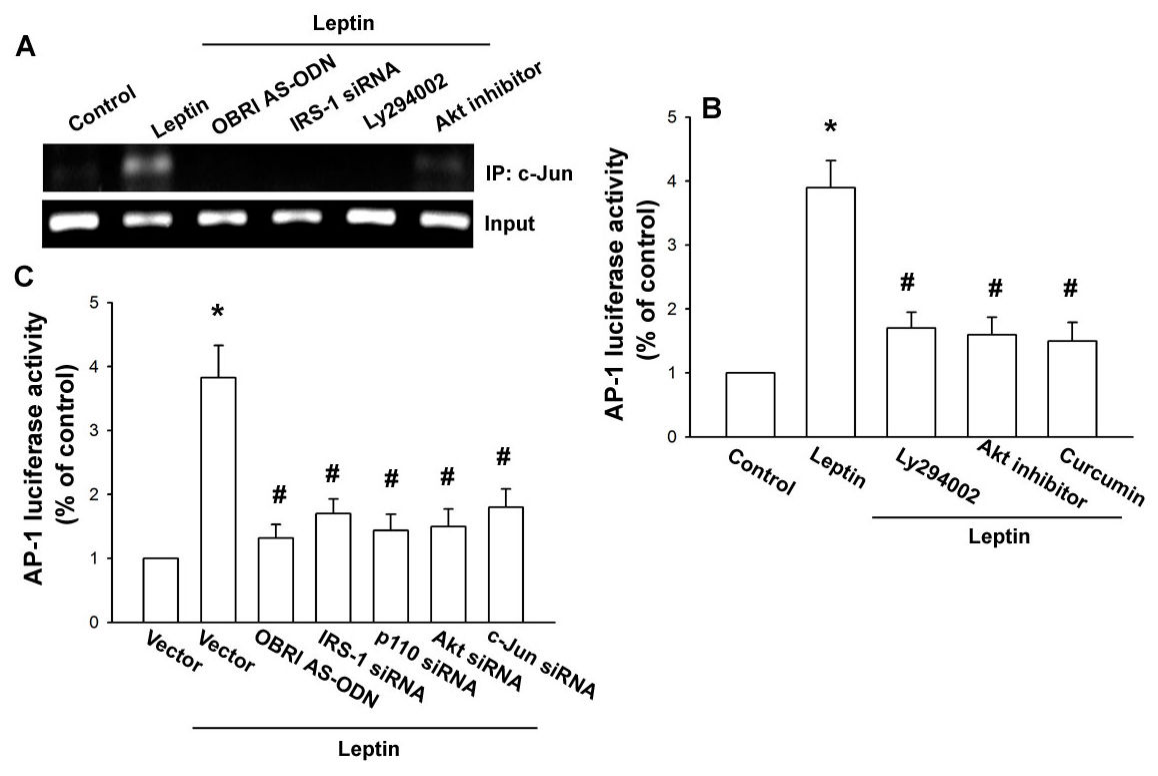
Leptin induces AP-1 activation through OBRl receptor/IRS-1/PI3K/Akt pathway. (A) OASFs were pretreated with Ly294002 and Akt inhibitor for 30 min or transfected with OBRl AS-ODN and IRS-1 siRNA for 24 h and then stimulated with leptin for 120 min, and chromatin immunoprecipitation assay was performed. Chromatin was immunoprecipitated with anti-c-Jun. One percent of the precipitated chromatin was assayed to verify equal loading (input) (n=4). (B and C) OASFs were transfected with AP-1-luciferase expression vector and then pretreated with Ly294002, Akt inhibitor, and curcumin or cotransfected with OBRl AS-ODN, IRS-1 siRNA, p110 siRNA, Akt siRNA, and c-Jun siRNA before incubation with leptin for 24 h. Luciferase activity was then assayed. Results are expressed as mean ±S.E.M. of four independent experiments. *: *p* < 0.05 as compared with basal level. #: *p* < 0.05 as compared with the leptin-treated group.

Furthermore, leptin increased the binding of c-Jun to the AP-1 (−312 to −39) element within the IL-6 promoter, as shown by a chromatin immunoprecipitation assay (Figure 6A). Binding of c-Jun to the AP-1 element was attenuated by OBRl AS-ODN, IRS-1 siRNA, Ly294002, and Akt inhibitor (Figure 6A). Furthermore, using transient transfection with AP-1-luciferase as an indicator of AP-1 activity showed that OASFs incubated with leptin increase in AP-1 promoter activity (Figure 6B). The increase of AP-1 activity mediated by leptin was reduced by OBRl AS-ODN, IRS-1 siRNA, p110 siRNA, Akt siRNA, and c-Jun siRNA, or Ly294002, Akt inhibitor, and curcumin (Figure 6B and C). These data indicated that leptin increased IL-6 production in OASFs via the OBRl, IRS-1, PI3K, Akt, and AP-1 signaling pathway.

## Discussion

OA is a heterogeneous group of conditions that are associated with defective integrity of articular cartilage. The chronic inflammatory process is mediated through a complex cytokine network. The factors responsible for initiating the degradation and loss of the articular tissues are not yet completely understood. The level of leptin in the synovial fluid of the joint has been recently shown to be much higher in OA patients compared to control individuals with no history of OA. In fact, leptin levels in the synovial fluid are strongly associated with the radiographic severity of OA [39], indicating a local effect of leptin in articular cartilage, and further suggesting that leptin levels in the synovial fluid could be used as an effective quantitative marker for detection of OA. First, we found that leptin promoted IL-6 production. In addition, the potentiation of IL-6 by leptin requires activation of the OBRl, IRS-1, PI3K, Akt, and AP-1 signaling pathway. These findings suggest that leptin acts as an inducer of inflammatory cytokines such as IL-6 and enhances the inflammatory response in OA. We previous reported that letpin induced IL-8 expression in human synovial fibroblasts through OBRl/IRS-1/PI3K/Akt/NF-κB pathway [40]. Therefore, both IL-6 and IL-8 are target proteins of the leptin signaling pathway that regulates the cell inflammatory response. In addition, OBR1/IRS-1/PI3K/Akt is common pathway in leptin-mediated pathogenesis in OA.

The leptin receptor belongs to the cytokine receptor superfamily [41]. Recent studies have demonstrated higher levels of leptin receptors OBRl and OBRs in the synovial fluid [29] and in the cartilage [32] of individuals with OA. It has been reported that leptin receptor expression is significantly elevated in advanced OA cartilage compared to minimally affected cartilage [2]. The results of this study showed that leptin increases the expression of OBRl but not that of OBRs. Furthermore, transfection with OBRl AS-ODN, but not with OBRs AS-ODN, antagonized the leptin-induced IL-6 production. These results suggest that OBRl is an upstream receptor in leptin-induced IL-6 release in human synovial fibroblasts.

Upon leptin binding, OBRl activates IRS proteins and stimulates the IRS-PI3K signaling pathway [35]. In the present study, we used IRS-1 siRNA and found that IRS-1 siRNA inhibited leptin-induced IL-6 production, indicating the possible involvement of IRS-1 in leptin-induced IL-6 release in synovial fibroblasts. It also has been reported that IRS-1 associates with p110, the regulatory subunit of PI3K, to regulate cell functions [42]. Pretreatment of synovial fibroblasts with PI3K inhibitors Ly294002 and wortmannin or transfection with p110 siRNA antagonized the increase of IL-6 expression mediated by leptin. A number of studies have demonstrated that leptin-induced PI3K activation leads to phosphorylation of phosphatidyl inositides and in turn activates the downstream main target Akt, which is an important protein kinase in regulating cell growth, differentiation, adhesion, and inflammatory reactions [36,43,44]. In this study, we demonstrated that the leptin-induced expression of IL-6 was inhibited by Akt inhibitor. This was further confirmed by the result that Akt siRNA inhibited the enhancement of IL-6 by leptin. Phosphorylation of Akt was found to be activated by leptin in synovial fibroblasts. These effects were inhibited by IRS-1 siRNA or Ly294002, indicating the involvement of IRS-1/PI3K-dependent Akt activation in leptin-mediated induction of IL-6.

A number of transcription factors, including NF-κB, CREB, NF-IL-6, and AP-1 box in the 5′ region of the IL-6 gene, have several binding sites [37,38]. Recent studies on the IL-6 promoter have demonstrated that IL-6 induction by several transcription factors occurs in a highly stimulus-specific or cell-specific manner [45]. The results of this study showed that AP-1 activation contributes to leptin-induced IL-6 production in synovial fibroblasts, and deletion of AP-1 site reduced leptin-mediated IL-6 promoter activity. Pretreatment of cells with AP-1 inhibitor (curcumin) or c-Jun siRNA also reduced leptin-increased IL-6 production. Therefore, the AP-1 binding site is important in leptin-induced IL-6 production. Furthermore, leptin increased the binding of c-Jun to the AP-1 element on the IL-6 promoter, as shown by the chromatin immunoprecipitation assay. Using transient transfection with AP-1 luciferase as an indicator of AP-1 activity, we also found that leptin induced an increase in AP-1 activity. In addition, transfection with OBRl AS-ODN, IRS-1 siRNA, p110 siRNA, Akt siRNA, and c-Jun siRNA or pretreatment with Ly294002, Akt inhibitor, and curcumin abolished leptin-increased AP-1 promoter activity. These results indicate that leptin may act through the OBRl receptor, IRS-1, PI3K, Akt, and AP-1 pathway to induce IL-6 activation in human OASFs. In addition to cytokine release, similar pathway has also been reported in the leptin induced IL-6 expression in microglia, which through OBRl/IRS-1/PI3K/Akt/NF-κB pathway [46], and in human synovial fibroblasts leptin promoted IL-8 production, which involved OBRl/IRS-1/PI3K/Akt/NF-κB activation [40]. Therefore, OBRl/IRS-1/PI3K/Akt signaling pathway may be a common signaling pathway in leptin-mediated gene expression.

OA is a degenerative joint disease characterized by cartilage breakdown, the formation of bony outgrowths at the joint margin (osteophytes), subchondral bone sclerosis, alterations to the joint capsule, and inflammation of the synovial membrane [47]. Inflammation of the synovium results in synovitis, which can occur in the early stages of OA [48]. Inflamed OA synovial tissue may release inflammatory factors or the damaged cartilage may trigger a systemic inflammatory response, leading to the release of inflammatory mediators by the synovium. Secreted inflammatory factors such as proinflammatory cytokines, therefore, are critical mediators of disturbed metabolism and enhanced catabolism of joint tissue involved in OA. In this study, we investigated the signaling pathway involved in leptin-induced IL-6 production in OASFs. The results showed that leptin increases IL-6 production by binding to the OBRl receptor and activating IRS-1/PI3K/Akt signaling, which enhances AP-1 transcription activity and leads to the transactivation of IL-6 production. On the other hand, leptin is significantly higher in synovial fluid of patients with OA and RA [29,30]. Therefore, assessing the expression of leptin may be a good marker for prediction of OA and RA.

## Supporting Information

Figure S1 **IL-1β induces IL-6 expression in human synovial fibroblasts.** OASFs were incubated IL-1β (10 ng) for 24 h. Total RNA was collected, and the expression of IL-6 was examined by qPCR assay. Results are expressed as mean ± S.E.M. of four independent experiments. *: *p* < 0.05 as compared with basal level.
(TIF)

Table S1 **Characteristic of OA patients.**
(TIF)
