# Supplementary figures and images for: Leptin Induces IL-6 Expression through OBRl Receptor Signaling Pathway in Human Synovial Fibroblasts
Source: PLoS One. 2013 Sep 27;8(9):e75551. doi: 10.1371/journal.pone.0075551 (PMC3785513; doi:10.1371/journal.pone.0075551)

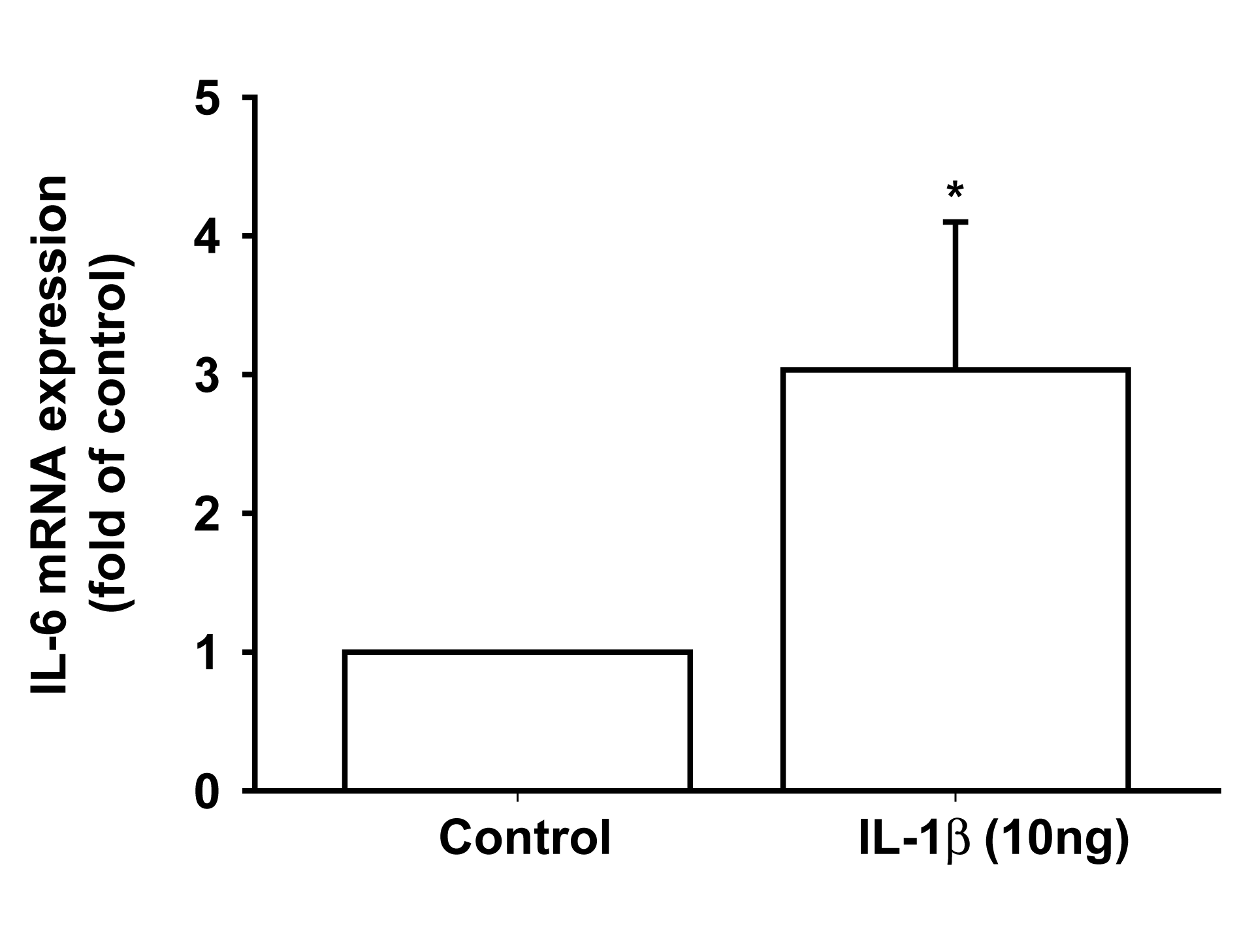

Supplement: Figure S1 — IL-1β induces IL-6 expression in human synovial fibroblasts. OASFs were incubated IL-1β (10 ng) for 24 h. Total RNA was collected, and the expression of IL-6 was examined by qPCR assay. Results are expressed as mean ± S.E.M. of four independent experiments. *: p < 0.05 as compared with basal level. (TIF) [file pone.0075551.s001.tif]

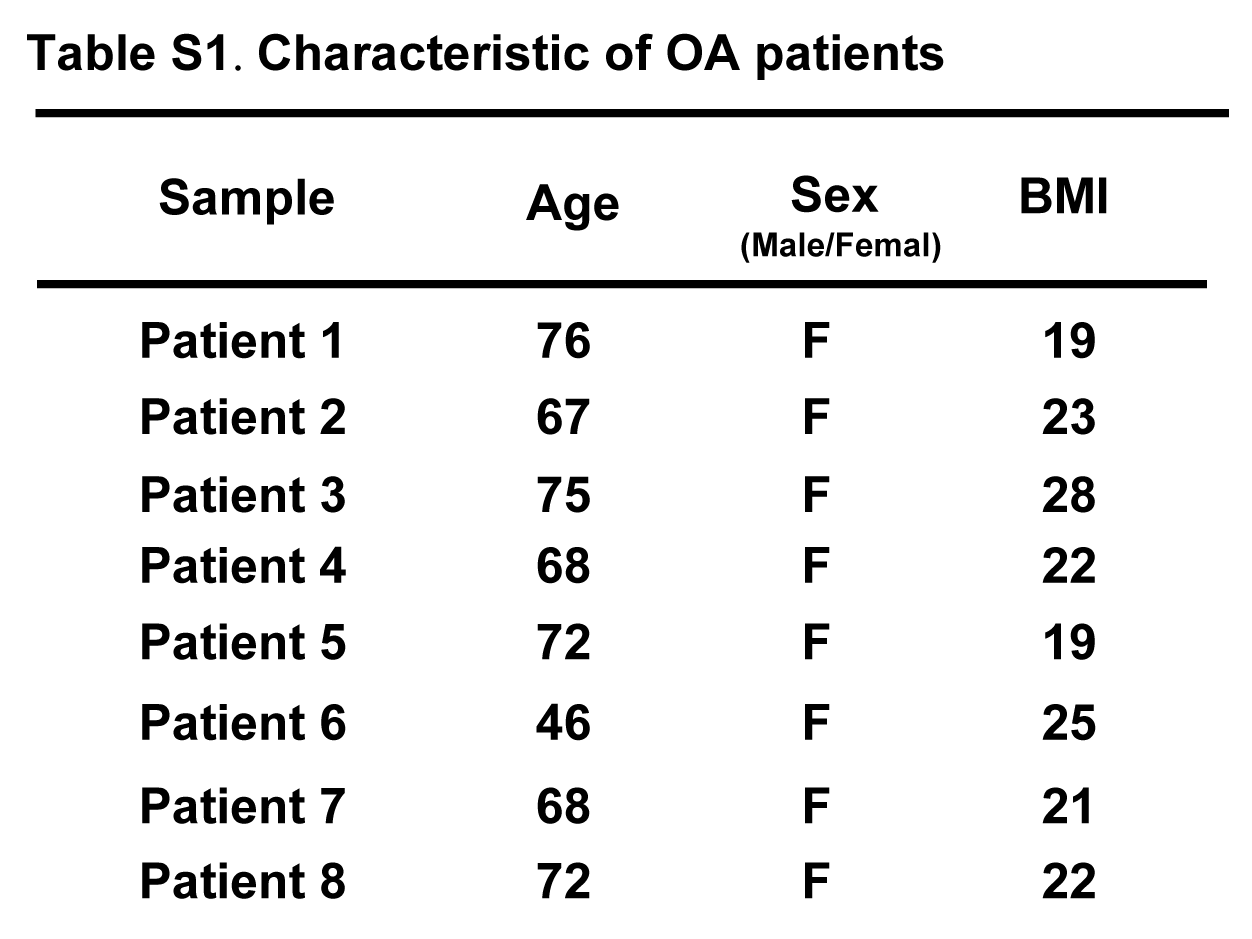

Supplement: Table S1 — Characteristic of OA patients. (TIF) [file pone.0075551.s002.tif]
